# A low-cost concurrent TSV test architecture with lossless test output compression scheme

**DOI:** 10.1371/journal.pone.0221043

**Published:** 2019-08-23

**Authors:** Young-woo Lee, Hyunchan Lim, Sungyoul Seo, Keewon Cho, Sungho Kang

**Affiliations:** Department of Electrical and Electronic Engineering, Yonsei University, Seoul, Korea; Huazhong University of Science and Technology, CHINA

## Abstract

As the traditional IC design migrates to three-dimensional integrated circuits (3D-ICs) design, new challenges need to be considered carefully to solve its reliability and yield issues. 3D-ICs using through-silicon-vias (TSVs) can have latent defects such as resistive open and bridge defects, which are caused by the thermal stress during the fabrication process. These latent defects lead to the deterioration of the electrical performance of TSVs caused by an undesired increase in the resistance-capacitance (RC) delay. For this reason, various post-bond test methodologies have been studied to improve the reliability of 3D-ICs. Cost reduction in these TSV test architectures is also currently being studied by decreasing various factors such as hardware overhead, test time, and the peak current consumption. Usually, a single test-clock-period is required to determine whether the test result contains the defective TSV. When the test result of any TSVs fails, we use another single test-clock-period to classify its defect type. In this paper, we propose a new TSV test architecture to transfer the combined test output of the test result and the specific defect type to the pad during the single test-clock-period. Our proposed test architecture also provides a reliable block-based concurrent testing to optimize the test time by dividing the die into concurrent blocks. The experimental results showed that our proposed test architecture could reduce the test time and the hardware overhead substantially by ensuring that the reasonable peak power consumption for mass production was reasonable without the test quality being adversely affected.

## Introduction

Three-dimensional (3D) integration technology is an emerging fabrication technique that vertically stacks multiple device layers by using through-silicon-vias (TSVs) [1, 2]. Such 3D stacked devices can provide a solution to scaling or to interconnection challenges, such as the technological limitations due to continued downscaling or the higher interconnect delays in nanoscale integrated circuits (ICs) [3, 4]. Theses multiple device layers are vertically bonded by wafer stacking or die stacking, and each layer in the three-dimensional integrated circuit (3D-IC) is connected using TSVs and microbumps. These layers serve as interconnections and provide power improvement, high performance, and high throughput bandwidth by reducing the wire length. TSV-based 3D-ICs offer various benefits such as high device density and flexible signal routing [5]. Several 3D integration technologies have been released, and many of these have been employed for the manufacturing prototypes and for the mass production of devices [6–8]. However, latent defects of various kinds can occur in TSVs due to thermal stress at any step during the TSV manufacturing process [9]. These TSV defects will ultimately lead to the deterioration of the electrical performance of TSVs; they can cause additional signal delays or voltage drops [10]. Consequently, the reliability of 3D-IC can be degraded by the latent defects in TSVs due to the thermal stress [11]. It clearly results in a decrease of the yield and performance of 3D-ICs. To overcome these drawbacks, many effective TSV test techniques and design-for-testability (DFT) solutions have been developed [12–18]. There are two basic ways for testing TSVs in 3D-ICs: pre-bond testing and post-bond testing. Pre-bond testing is performed before the TSVs are bonded; this process detects various TSV defects that can occur during TSV fabrication or manufacturing. This process is performed before wafer bonding or die bonding; therefore, the localized defects in each TSV can be analyzed during the TSV manufacture. Post-bond testing is conducted after stacking the dies having more than two layers. The post-bond test allows us to detect the device’s functional defects for 3D-ICs caused by the misalignment of TSVs, or by the high temperatures and pressures after the stacking process [19–21]. It is important to detect TSV defects at an early stage. The TSV test is essential for improving the quality and yield of 3D-ICs during mass production; stacked layers are completely discarded if there is only one defective layer in the 3D-IC [22–24]. It is profitable for semiconductor companies to reduce their test cost for mass production. The test cost is closely related to the test time because a short test time can reduce the test cost of 3D-ICs. In addition, power consumption may reach a peak during TSV testing, and there is a perceived trade-off between the peak power consumption and the cost of automatic test equipment (ATE). Consequently, given peak power consumption constraints, it is important to reduce the test time of 3D-ICs without degrading the test quality.

This paper proposes a cost-effective TSV testing architecture for post-bond constraint-based testing. Various market forces have compelled companies to produce high-density TSVs that use the 3D-IC technologies. The number of TSVs used in 3D-ICs will continue to increase, which will place a growing demand on interconnected bandwidth levels in system-on-chip (SoC) architectures. With the increase in test times and hardware overheads, the test cost of the previous TSV test architectures becomes directly proportional to the number of TSVs in the 3D-ICs. Our new TSV test architecture has a shorter test time and a lower hardware overhead than previous TSV test architectures, with maintaining the equivalent test quality and the appropriate peak current consumption for mass production. It is also possible to provide the optimized wire overhead by using the TSV block partition method.

## Background

### Various TSV defect types

**Fig 1:**
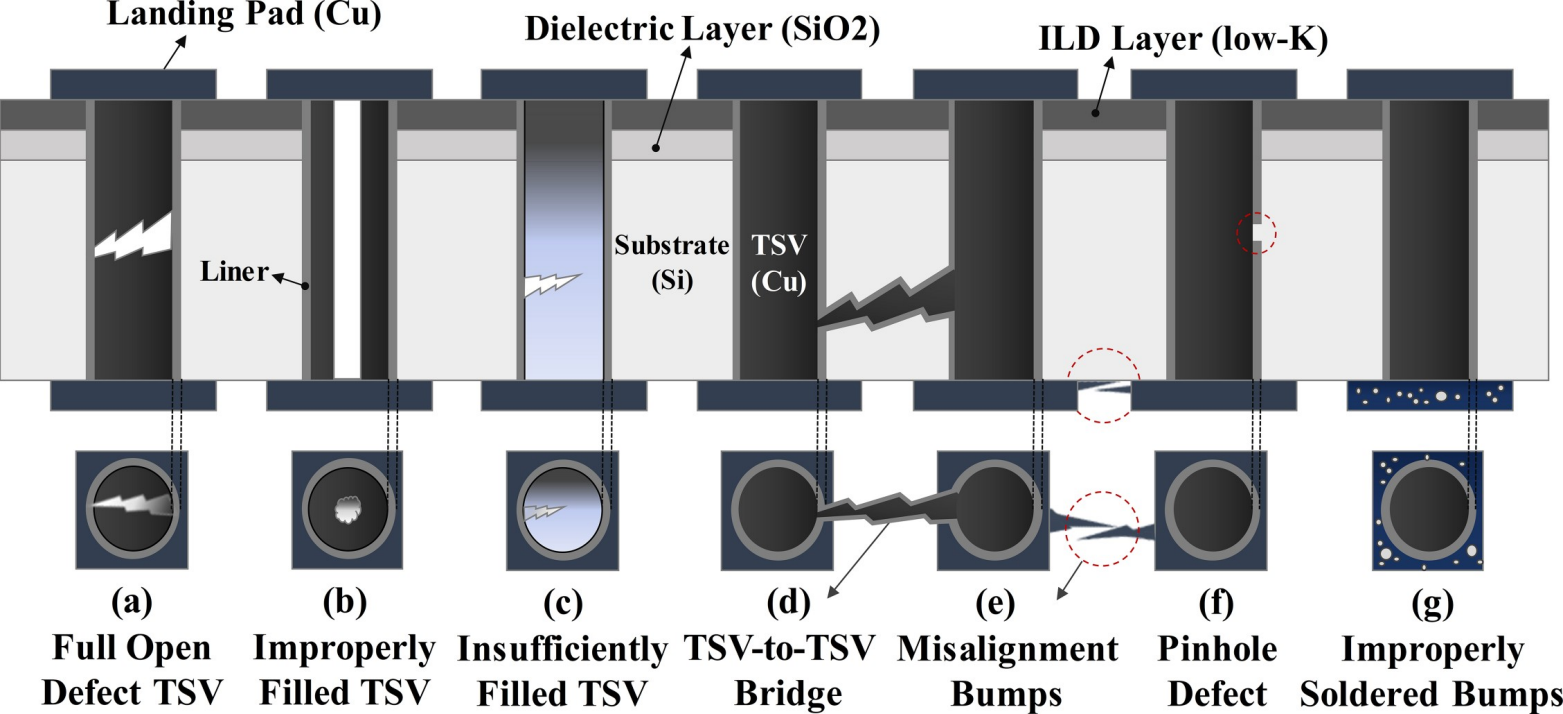
Various TSV defects.

TSV-based 3D-ICs have many TSVs between their layers and, various TSV defects can occur at any stage during the TSV manufacturing process as shown in Fig 1 [19–21]. In a full-open TSV defect (Fig 1(A)), the signal cannot be transmitted from the previous die to the next die. The second defect is an improperly filled TSV (Fig 1(B)) or an insufficiently filled TSV defect (Fig 1(C)). The TSV is supposed to be fully filled with void-free copper (Cu). Violations of this condition can lead to resistive open defects. The third defect is a TSV-to-TSV bridge defect in which the TSVs are internally shorted together in the substrate by the Cu leak (Fig 1(D)), or the landing pads and bumps are in contact because of misalignment (Fig 1(E)). In pinhole defects (Fig 1(F)), a TSV is exposed to the silicon (Si) substrate. The final defect occurs when the TSVs are fault-free but the bumps are improperly fabricated, which lead to intermittently resistive open defects (Fig 1(G)). This fault can be detected only by repeating the tests. The various TSV defects can be divided into two general classes: resistive open defects and TSV-to-TSV bridge defects. All these TSV defects affect the yield of 3D-ICs; therefore, it is important for strategic cost reduction of 3D-ICs to conduct preliminary inspections for both categories of TSV defects.

### Test modeling of TSVs with resistive open defects

There are two types of resistive open defects in TSVs: fully open and partially open. Fully open resistive TSVs must be replaced with fault-free TSVs by performing a TSV repair process. Partially open resistive TSVs are improperly or insufficiently filled, or contain micro-voids. In such cases, the usable TSVs can be sorted by resistive open defect testing. The electrical modeling of pre-bond TSVs can be modeled by a small capacitor, and post-bond TSVs can be represented by various equivalent circuit models such as *RLC* model, *RLC* π-model, *RLCG* model and so on [25–28]. However, we use the simplified circuit model of the TSV to test the resistance-related delays across the TSV during mid-band frequency tests, which is based on the same assumptions as described in previous works [15–18]. Each post-bond TSV has its own internal resistance and capacitance, depending on the manufacturing conditions. From these two parameters, we can calculate the *RC* delay time across the defective TSVs [29, 30]. The TSV can be modeled by a resistance *R*, an inductance *L*, and a capacitance *C*. However, the inductance *L* is often neglected if TSVs are not used for transmitting high-frequency analog signals [31]. The field-effect transistor (FET) is generally used as a driver on Die1 or as a receiver on Die2 for TSV testing [15–18]. The voltage across the TSV is calculated as follows:

$$V_{TSV}=\frac{R_{T}}{R_{S}+R_{TSV}+R_{T}}\times V_{S}$$ (1)

where *R*_*TSV*_ is the resistance of the TSV, *R*_*S*_ is the on-resistance of the driver, *R*_*T*_ is the on-resistance of the receiver, and *V*_*S*_ is the supply voltage.

The devices in 3D-ICs have their own operating speeds and critical paths, and the TSVs are not supposed to have any effect on this period. The resistance of each TSV is indirectly calculated by measuring the voltage across the TSV. Among the TSVs with partially open defects, the usable ones are salvaged by selecting an appropriate reference voltage for the comparator. The voltage is selected depending on the speed characteristics of the devices used in the 3D-ICs. The TSV test architecture based on the voltage divider structure such as previous works [15–18] is designed for testing the resistance-related delays across the TSV as part of the at-speed test. For yield improvement, it is important to catch the soft faults in the defective TSVs by characterizing the resistance of the specific TSV with partially open defects. The yield of the 3D-ICs can be improved by resetting the reference voltage for detecting the soft fault in TSVs with the resistive open defect; the resetting needs to be performed on the basis of the characterized TSV resistance value during the silicon debugging; depending on the timing specifications of 3D-ICs.

### Test modeling of TSVs with TSV-to-TSV bridge defects

**Fig 2:**
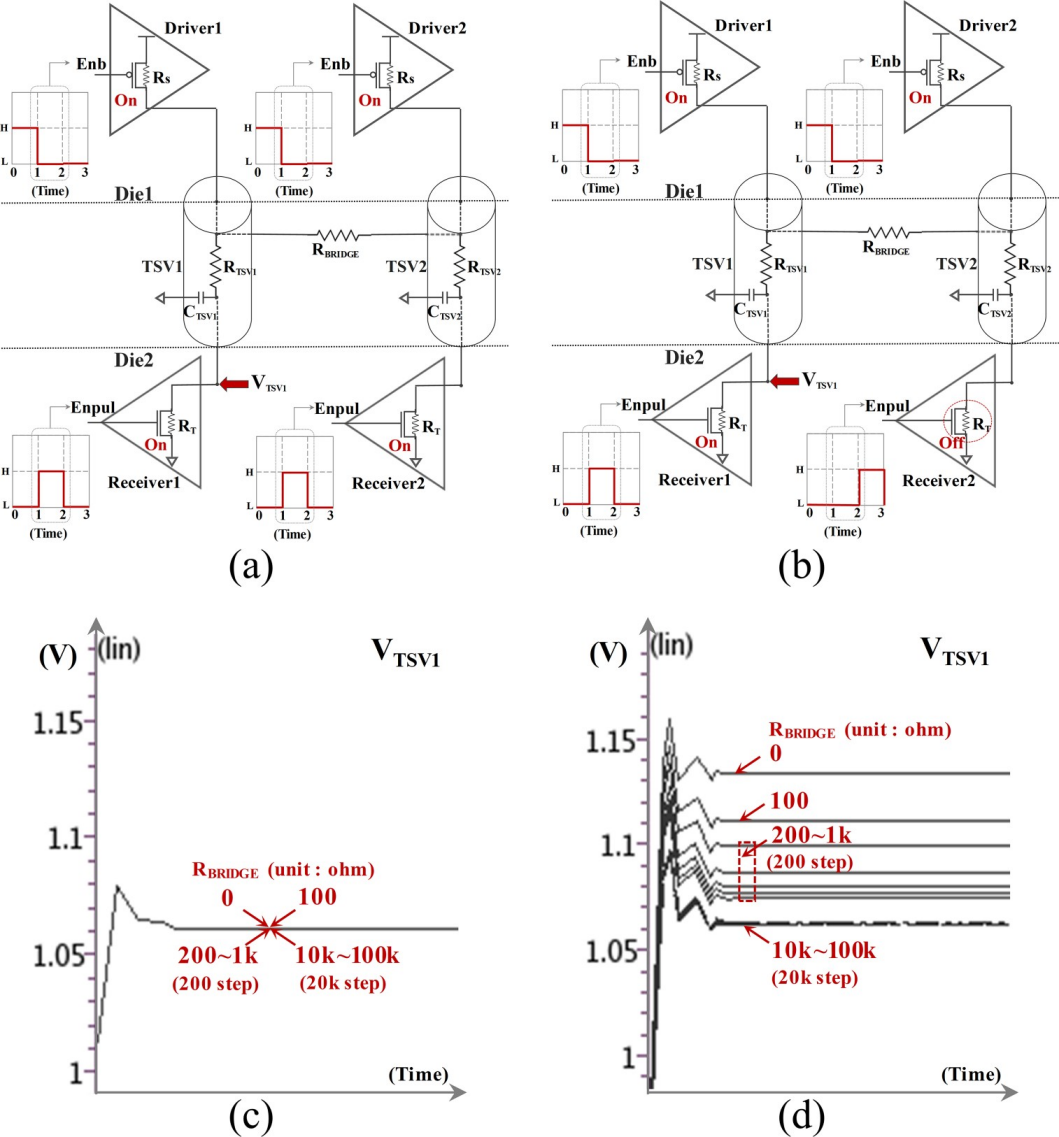
Test conditions for detecting TSV-to-TSV bridge defects: (a) typical test designs; (b) recommended test designs; (c) *V*_*TSV*_ in case of (a); (d) *V*_*TSV*_ in case of (b).

Fig 2(A) represents the typical test design based on the voltage divider structure. Unfortunately, the typical test modeling for resistive open defects cannot sort out TSV-to-TSV bridge defects because the TSVs are based on the same test conditions. If the two TSVs are shorted together, the voltage across the TSV may be affected by the low-resistance connection between the two points. However, if each resultant resistance has the same value, the voltage at each connected point will be equal regardless of the low-resistance connection. Therefore, as shown in Fig 2(C), the voltage across the TSV (*V*_*TSV*_) remains constant regardless of the resistance between the two TSVs *(R*_*BRIDGE*_) [16]. Therefore, it is important to detect the TSV-to-TSV bridge defects for creating a different resultant resistance at each connection point. This condition can be implemented by sequentially testing TSVs by controlling the driver or the receiver. To detect the TSV-to-TSV bridge defects, the test models must meet at least one of the two following conditions. First, the driver supplies a voltage to only one TSV during a single test cycle. Second, only one receiver resistance (*R*_*T*_) must be used or enabled per test cycle (Fig 2(B)). Under any one or both conditions, *V*_*TSV*_ can be altered by depending on *R*_*BRIDGE*_ by affecting different voltages at each connected point between the two TSVs. Fig 2(D) presents the voltage profiling graph under the second test condition; note that both the voltage level across the TSV and the shape of the graph can be changed in case the first condition or both conditions are implemented. The voltage across the TSV is measured by enabling only the receiver resistor (i.e., *R*_*T*_) of the currently selected TSV; the receiver resistances are controlled by turning the FETs on or off. To increase the test reliability and 3D-IC yield, these two test conditions need to be considered during the early stage of the device design process. These are very important for enabling the concurrent TSV test in 3D-ICs.

## Proposed test architecture

In this section, we introduce our block-based concurrent TSV test architecture, which can transfer the combined result by using the test output compression scheme. It is designed for enabling the verification of the resistance-related delays of TSVs in 3D-ICs for inspecting the resistive open and TSV-to-TSV bridge defects based on the voltage divider structure. Unlike previous test architectures, the proposed test architecture can effectively reduce the hardware overhead and the test time by ensuring appropriate current consumption for mass production and by maintaining the equivalent test quality.

### Motivation

**Fig 3:**
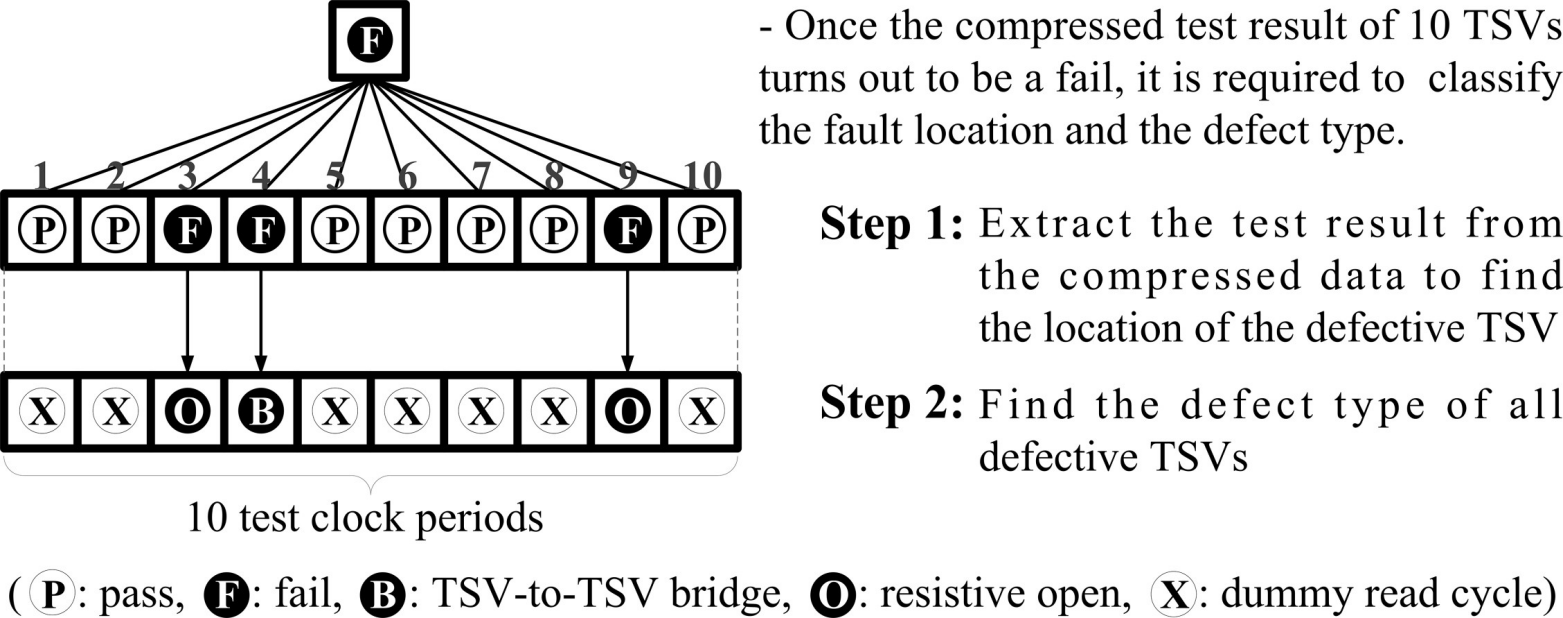
Previous extraction process of the compressed test result.

In general, previous test architectures use the parallel and/or output compression test technique to optimize the test time of the post-bond testing. The parallel test method can test selected TSVs together for time-saving. The output compression is used to judge the defective TSV among the selected TSVs, which act as the parallel test group during the single test-clock-period. However, there are some drawbacks for the parallel test method. The first drawback of this method is its inability to detect TSV-to-TSV bridge defects such as previous works [13–15]. The hardware overhead costs in the architectures used in theses previous works are relatively high. As stated in [21], a 3D-stacked memory manufacturer mentioned that the fast Cu leak monitor method is essential for maintaining a high quality and the Fab process control. To improve the manufacturability and reliability of 3D-ICs, it is important to consider the detection of the TSV-to-TSV bridge defects and the open defects in the future. Previous works [17, 18] were designed to overcome these shortcomings. However, the obvious problem of the output compression scheme still remains that additional test-clock-periods are used for determining the defective TSV and its defect type whenever the compressed test result failed. In this case, two test-clock-periods are required for every TSV in a parallel test group to transfer the test result and the defect type, as described in Fig 3 (i.e., 2 × the number of TSVs in the parallel test group). Another drawback of the architecture given in [17] is that the test time is highly affected by the TSV failure rate. The probability of the existence of a defective TSV in the parallel test group rises with an increase in the TSV failure rate. Then, the number of parallel test groups perceived as failed tests also increases, and the total test time deteriorates in proportion to the increment of the additional test-clock-periods required for determining the defective TSV and its defect type in all groups (i.e., 2 × the number of TSVs in the parallel test group × the number of parallel test groups perceived as tests failed). When the number of TSVs in 3D-ICs is high, the *n* value in the parallel test group is set to a large number in the *n* × *n* matrix in the previous work [17]. The large *n* value, which specified the number of TSVs in the parallel test group, gives the worst test time depending on the TSV failure rate.

To overcome all these limitations and drawbacks of previous architectures, we propose a block-based concurrent TSV test architecture, which can transfer the combined test output of both the test result and the defect type in parallel during the single test-clock-period. We also consider a simultaneous test using window comparators, which can detect TSVs with resistive open and bridge defects at the same time. In addition, our proposed model has little or no effect on the total test time depending on the TSV failure rate with providing the reasonable peak current consumption for mass production. As already mentioned, the number of TSVs in 3D-ICs is constantly increasing and directly influences the total execution test time, as well as the total hardware overhead in previous test architectures. The increase in the test times and hardware overheads as functions of the TSV number is much slower in our proposed TSV test architecture as compared with the increase in the previous TSV test architecture.

### Constraints and considerations for concurrent test

High-density TSVs on the current 3D-ICs enable us to use the DFT techniques for added cost savings because these TSVs use the concurrent test method, which significantly reduces the test times. The proposed test architecture is based on the concept of the simultaneous testing of multiple TSV blocks, regardless of whether the TSV distributions are uniform or nonuniform. To perform the concurrent test, some aspects of 3D-ICs and ATE need to be considered. The 3D-IC devices need to be designed such that they can be tested concurrently. Some problems that can complicate the concurrent test include high power consumption and the inability to detect a TSV-to-TSV bridge defect when testing multiple TSV blocks concurrently. In addition, some issues need to be considered for ATE, such as the drive strength of the digital channel for resource sharing and the maximum current output of the device power supply (DPS).

**Fig 4:**
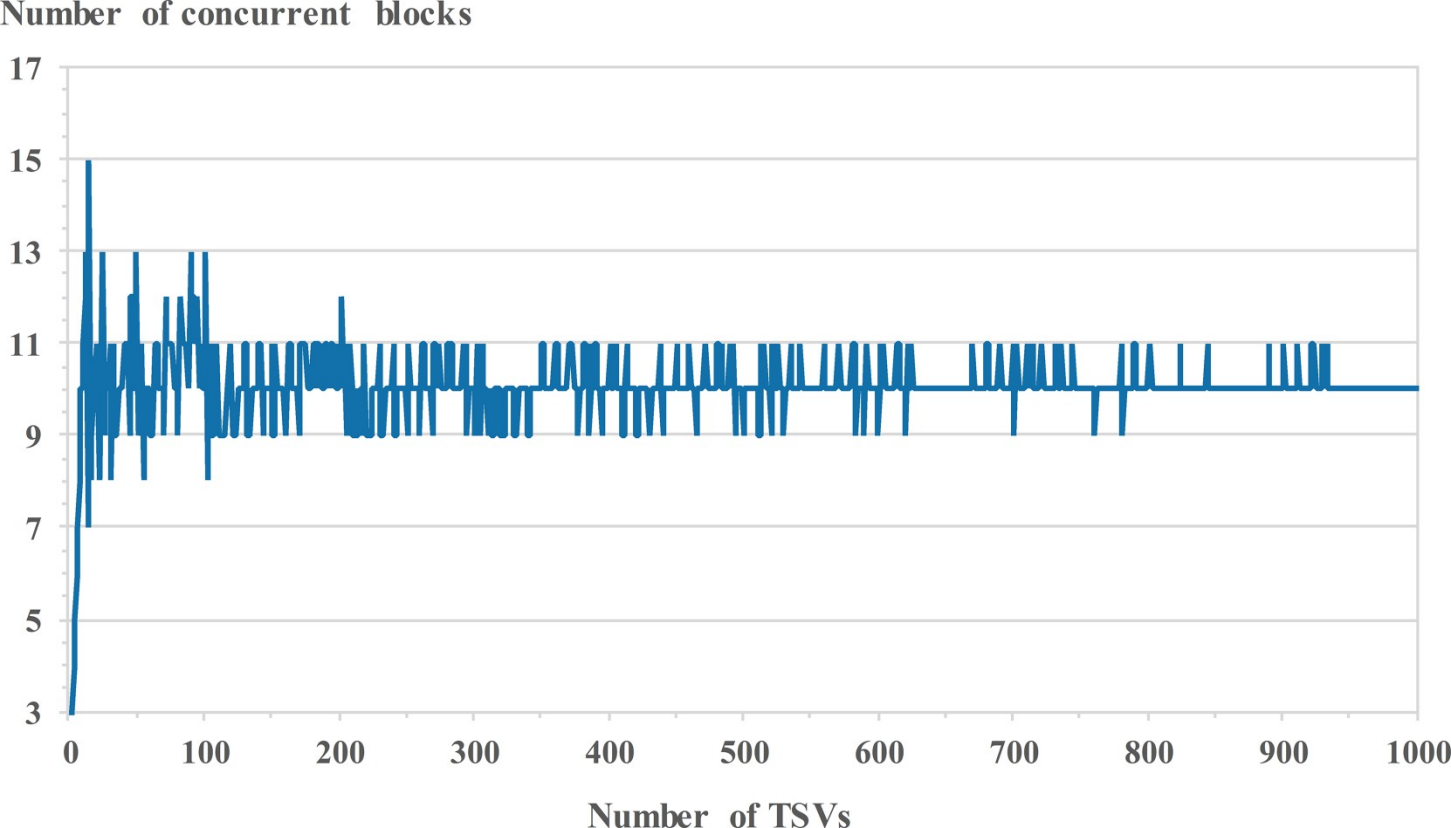
Optimized *N*_*Block*_ as functions of *N*_*TSV*_.

For the concurrent tests, the test time can be drastically reduced based on the number of concurrent test blocks. To maximize the concurrent test efficiency, we need to determine the maximum number of concurrent TSV blocks under power consumption constraints, depending on the maximum current output of the DPS being used for mass production. The DPS having a high pin count is most widely used in the industry; this DPS is generally applicable to current outputs below 800 mA [17]. Other ATE hardware limitations are also critical determinants for maximizing the number of concurrent TSV blocks, such as the drive strength of the digital channel for resource sharing and the available number of digital channels for TSV testing. The drive strength of the most widely used digital channel is approximately 50–100 mA per channel [32]. The number of TSVs in each concurrent TSV block (*N*_*TSV_Block*_) can be obtained by dividing the total number of TSVs (*N*_*TSV*_) by the number of concurrent TSV blocks (*N*_*Block*_). To clarify the required test conditions for enabling the concurrent TSV test architectures, we used the following inequality:

$$\frac{N_{TSV}}{N_{TSV\_Block}}\times Peak\_I_{TSV}\leq Max\_I_{DPS}$$ (2)

where *Peak_I*_*TSV*_ is the peak current consumption of the specific TSV test and *Max_I*_*DPS*_ is the maximum current output of DPS. Consequently, it is important to determine *N*_*Block*_ by considering *Max_I*_*DPS*_ of ATE. After obtaining *N*_*Block*_, the total test time is linearly decreased to the optimized value as an ideal case; the optimized value was obtained by dividing the original test time by *N*_*Block*_. However, a single TSV result in each concurrent TSV block should be collected for classifying the fault location and the defect type of all defective TSVs; this will help improve the yield of 3D-ICs by repairing and analyzing defective TSVs when the merge result of the concurrent test fails. The additional test time increased as the number of TSVs in the parallel test group for each concurrent test group perceived as a failed test. Therefore, it is not easy to determine the best *N*_*Block*_ because the actual total test time can be calculated by the equation *N*_*TSV_Block*_ + (*N*_*Block*_ × *N*_*Fail_Group*_), where *N*_*Fail_Group*_ is the number of concurrent test groups perceived as failed tests. The total test time depends on both *N*_*Block*_ and *N*_*Fail_Group*_; therefore, we simulated the fault model experiment 10,000 times as functions of *N*_*TSV*_ by using an exhaustive search method. Recent TSV technologies have a TSV failure rate between 0.005% and 5% [33, 34]. The used defective probabilities in our simulation were 0.5%, which gives a 1% probability for the total TSV defects including resistive open and bridge defects, as described in recent research paper [13, 17]. Fig 4 shows the best *N*_*Block*_ of each TSV number among 60 concurrent blocks up to 1,000 TSVs. The best *N*_*Block*_ converges into ten concurrent blocks for the fastest test time condition. Consequently, it is not necessary to consider *N*_*Block*_ under conditions of 1,000 TSVs. If the number of TSVs in 3D-ICs exceeds 1,000 TSVs, the best *N*_*Block*_ below 10,000 TSV can be set to a value between 10 and 12 blocks. The concurrent TSV test architecture is designed based on this best *N*_*Block*_, which was simulated by considering the ATE hardware limitations. Finally, it is impossible to detect TSV-to-TSV bridge defects when the shorted TSV pairs are simultaneously tested under the same test conditions as described in Section 2.3. To prevent this problem occurring during the concurrent test, a single TSV of each concurrent test block is tested from the upper left to the lower right in parallel. The enabled TSV of each concurrent test block can be physically separated under this condition; therefore, it is impossible for the enabled TSVs to be internally shorted together during the concurrent test.

### TSV block partition method for concurrent test

In general, most previous test architectures share the test and detection blocks during the post-bond testing. To decrease the test time and hardware overhead, the concurrent TSV test architecture also needs the individual shared test and detection blocks for each partitioned TSV block. Therefore, the wire overhead is increased by connecting each TSV with the shared test or the detection block. To minimize this drawback, we introduce the TSV block partition method for effectively providing a solution for the optimized wire overhead. The TSV distribution in 3D-ICs can be divided into two categories depending on the device type. The TSV distribution of the 3D stacked memory is uniformly placed, but the TSV placement of the 3D-stacked SoC exhibits a nonuniform distribution. The TSV block partition method for uniform distribution is very simple because each TSV block can be identically divided into *N*_*Block*_; however, this is not the case with nonuniform distribution. In contrast, it is difficult to split the nonuniform TSV distribution into suitable partition blocks. To provide the partitioning method for the concurrent test, we used the clustering algorithm. The clustering algorithm is a common analytical method used for data mining and machine-learning; this algorithm can be used to categorize clusters into groups. The *k*-means algorithm is one of the most suitable algorithms to sort TSVs equally into 10 groups in clustering algorithms, where *k* is the number of groups. To implement an effective solution for partitioning the concurrent TSV blocks, we introduce the *k*-means clustering algorithm with the same size constraints as those given below.

1. The algorithm randomly selects *k* TSVs among TSVs (i.e., *N*_*TSV*_) as the *k* initial concurrent block centers.
2. Each TSV is assigned to a closed initial concurrent block center if the number of TSVs in that concurrent block does not exceed *N*_*TSV_Block*_, which is based on the Euclidean distance between each TSV and each initial concurrent block center.
3. Each initial concurrent block center is reassigned as the average of TSVs in each concurrent block.
4. Steps 2 and 3 are repeated until no TSVs change the concurrent block anymore.

**Fig 5:**
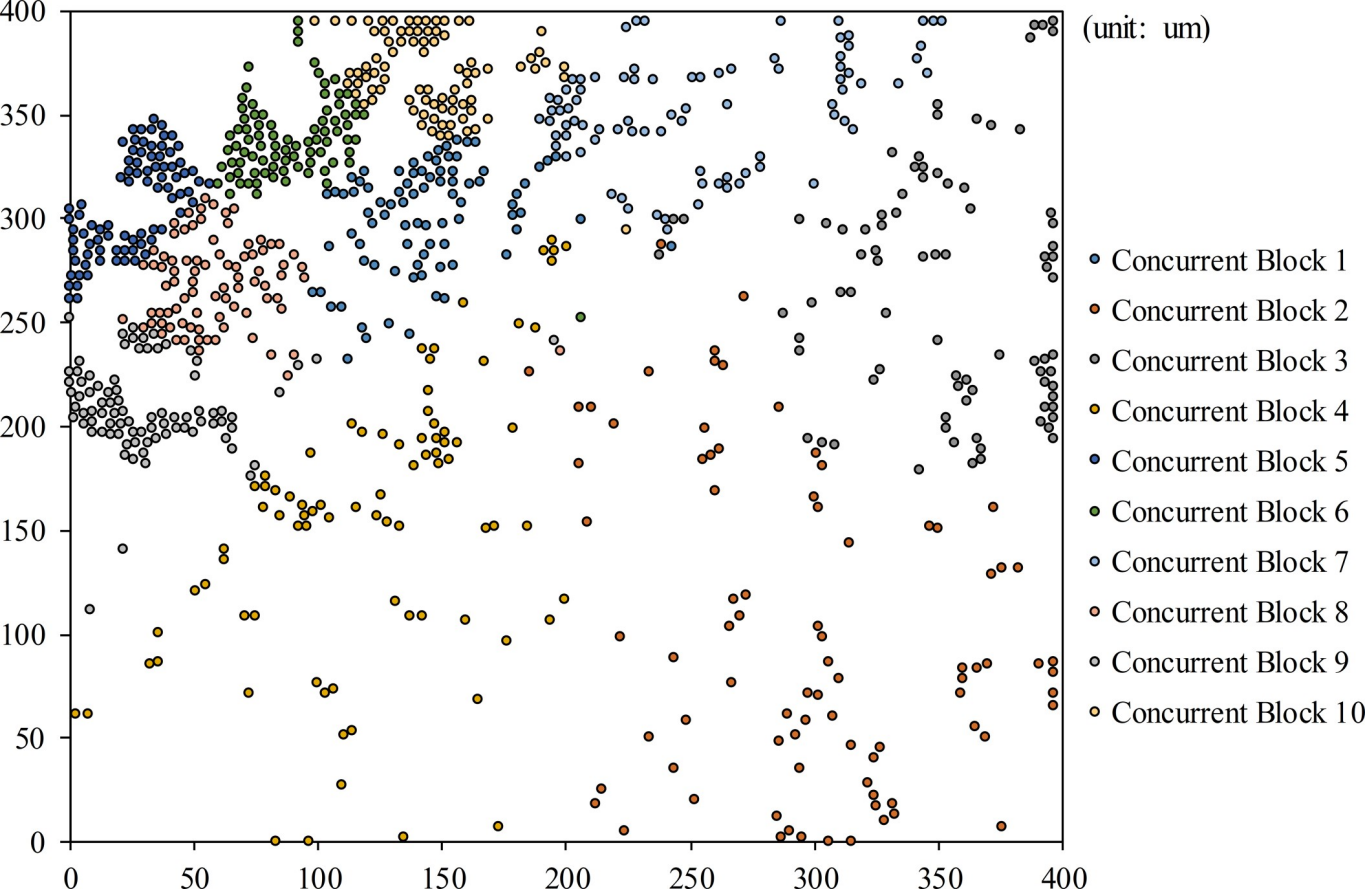
Result of the TSV block partition method in case of actual TSV distribution of the AES device.

Fig 5 shows the result when the TSV block partition method for the concurrent test is applied to the actual TSV distribution of the Advanced Encryption Standard (AES) device, which contains 837 TSVs [35]. For the proposed concurrent TSV test architecture, *N*_*Block*_ is always 10 for 1,000 TSVs as described in Section 3.1. The *k* value must be the same as *N*_*Block*_, and *N*_*TSV_Block*_ can be obtained by dividing *N*_*TSV*_ by 10. This approach improves performance and reduces the wire overhead of 3D-ICs by optimizing the wire length between each TSV and the shared test or the detection block.

### Concurrent TSV test architecture

**Fig 6:**
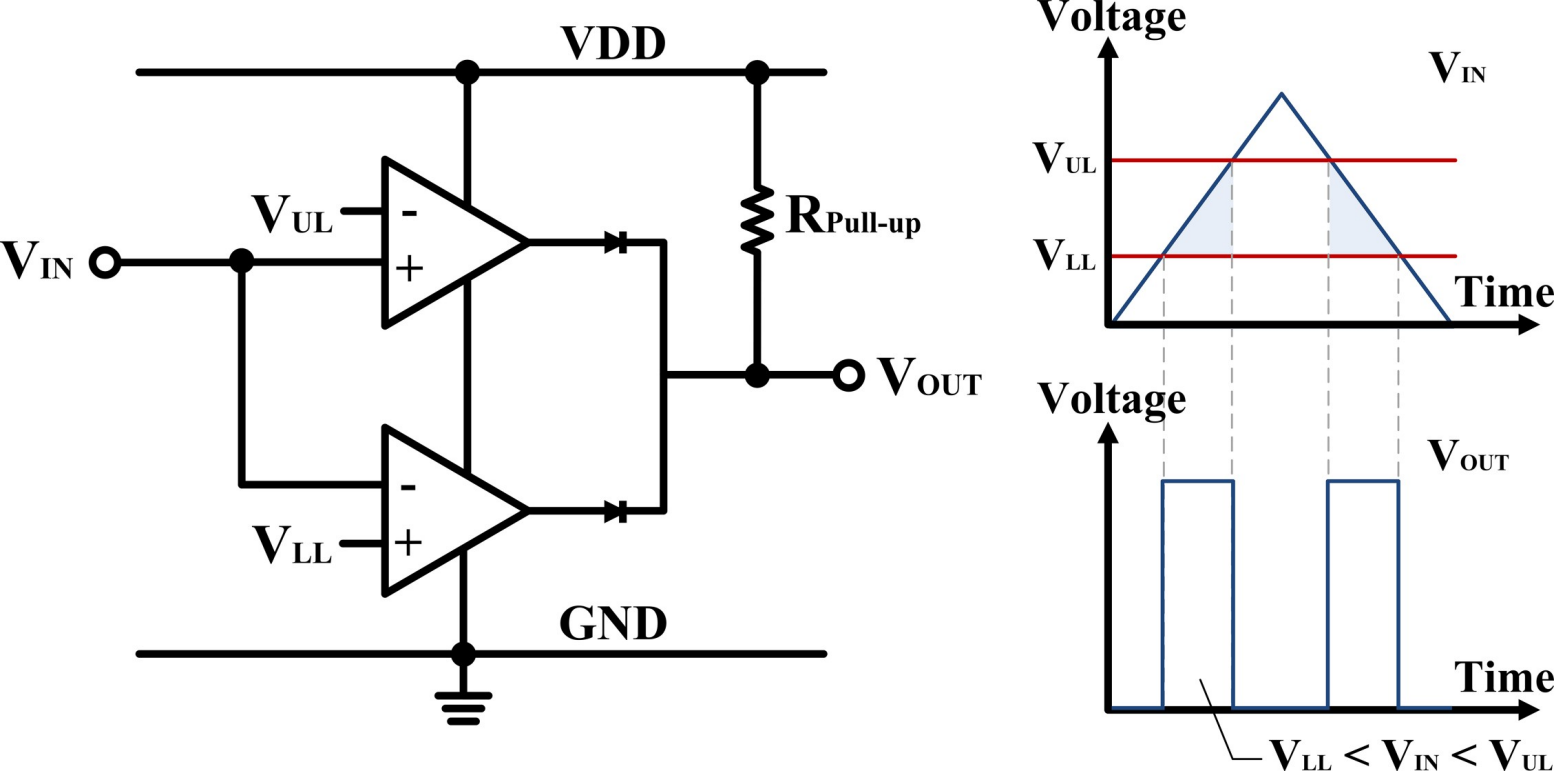
Window comparator circuit.

The proposed test architecture is composed of seven main parts: the voltage driver, voltage divider, concurrent test controller, defect detector, test result analyzer, test clock controller, and output compression/decompression circuits. The pMOS transistors on Die1 are used as voltage drivers, and the pMOS and nMOS transistors on Die2 are implemented as voltage dividers to divide the voltage across the TSV. The concurrent test controller can selectively activate the voltage divider of a single TSV for each concurrent TSV block during the one test-clock-period by using pulse-transfer flip-flops. The defect detector consists of two pairs of voltage comparators—one for detecting open defects and the other for detecting TSV-to-TSV bridge defects—as the window comparator functionality. When the input voltage (V_IN_) is greater than the reference voltage (V_REF_), the output of the non-inverting voltage comparator saturates towards the supply voltage level (i.e., VDD). In contrast, the output changes the state and saturates at the ground voltage level when V_IN_ is less than V_REF_. In case of the inverting voltage comparator, it operates in the opposite way. Previous works based on the voltage divider structure also use a single comparator or two comparators with logic circuits as the defect detector. To optimize the hardware overhead of the defect detector, our test architecture uses the window comparator, which is basically comprised of the non-inverting and the inverting comparators combined in the single comparator stage as described in Fig 6. The window comparator detects the input voltage levels that are within the window of voltages between the upper limit level (V_UL_) and the lower limit level (V_LL_). When V_IN_ is higher than V_UL_ or less than V_LL_, the window comparator’s output returns the low state. In contrast, its output is in a high state when the V_IN_ level lies between V_UL_ and V_LL_. In case of the receiver resistor (i.e., *R*_*T*_) control method given in [16–18], the voltage level of the resistive open defects is below the lowest voltage level of the fault-free TSVs, and the voltage level of the TSV-to-TSV bridge defects is higher than the highest voltage level of the fault-free TSVs. Therefore, using the window comparator as the defect detector can simultaneously detect the TSV with resistive open and TSV-to-TSV bridge defects without both XOR gates and multiplexers, as compared with [17, 18]. As the fifth main part of the proposed test architecture, the test result analyzer judges the test results of all enabled TSVs, and it can be implemented by using the 10-input AND gate because *N*_*Block*_ is always set to 10 concurrent blocks in conditions below 1,000 TSVs as described in Section 3.1. Finally, the output compression/decompression circuits and the test clock controller are used to provide high test quality and analysis for the TSV repair and the Fab process control when the test result analyzer detects any TSV defect. This block consists of an active low tri-state buffer with two 10-bit parallel-in-serial-out (PISO) shift registers by controlling two multiplexers, which are synchronous with the free-running test-clock-period (*ClkB*).

**Fig 7:**
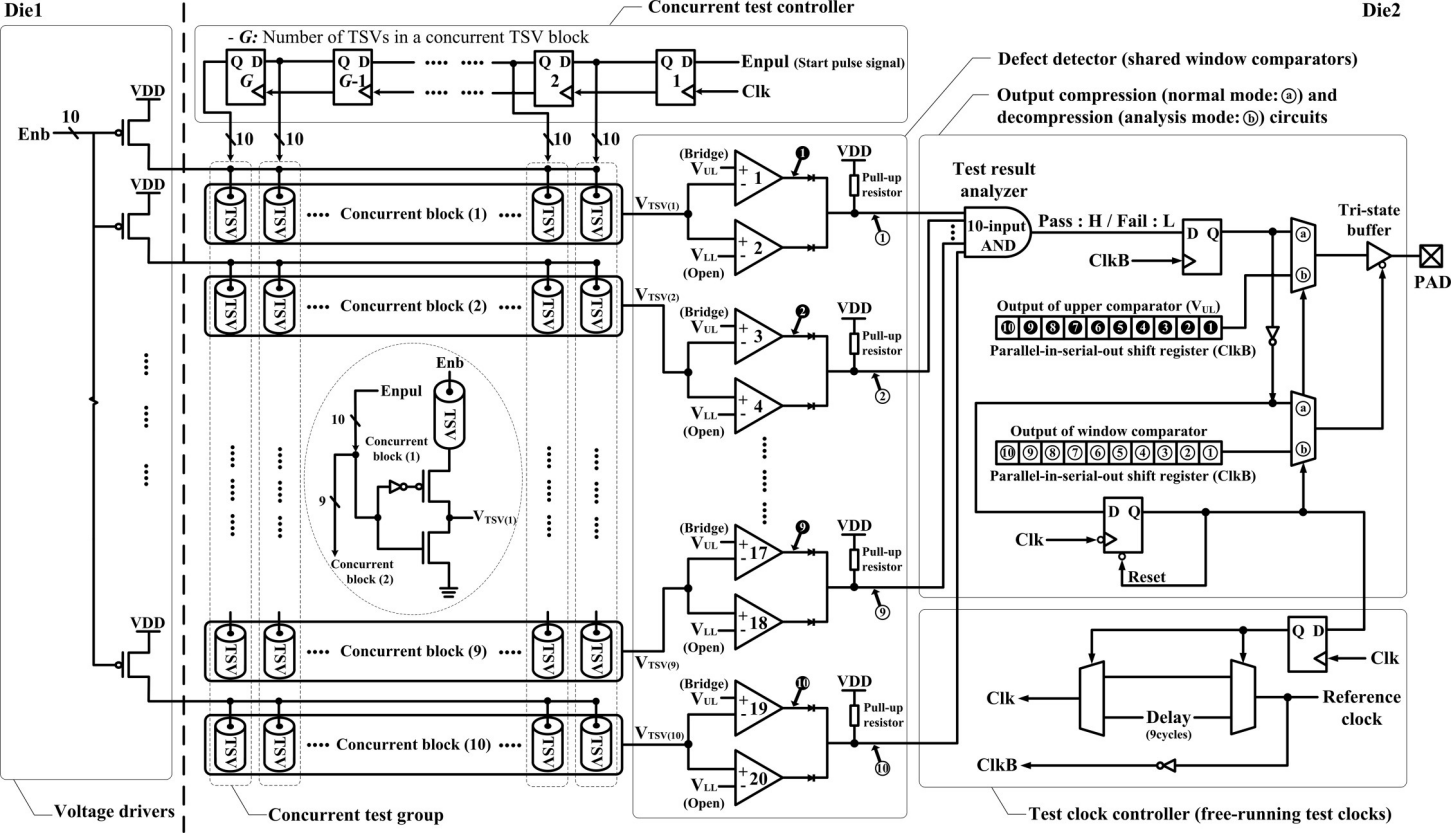
Proposed concurrent TSV test architecture.

**Fig 8:**
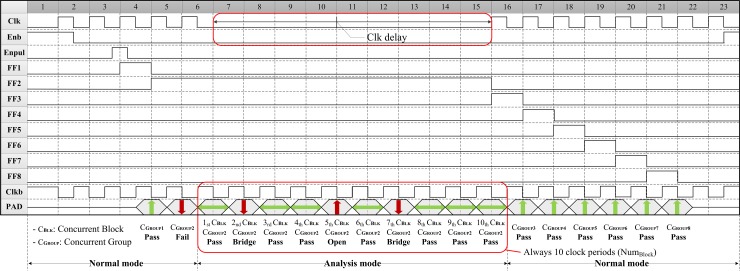
Example of the timing diagram of the proposed test architecture (*N*_*TSV*_: 80, *N*_*Block*_: 10, *N*_*TSV_Block*_: 8).

Fig 7 shows the overall block diagram of the proposed concurrent TSV test architecture. To induce the TSV test mode, the pMOS transistors connected to each TSV are turned on by using a high-to-low transition signal (*Enb*). Next, the pMOS and nMOS transistors are turned on by using the pulse signal (*Enpul*), which is generated only once. This enables the voltage dividers of the first TSV of each concurrent TSV block connected in parallel during the test cycle. When the pMOS and nMOS transistors are turned on, the voltage between the two transistors is calculated in accordance with the voltage division rule. The emerging output voltage across the TSV depends on the on-resistances of transistors and a TSV resistance. The *V*_*TSV*_ of the enabled TSV of each concurrent TSV block is conveyed to the shared window comparator, called the defect detector. Then, all the outputs of each defect detector are simultaneously conveyed to the test result analyzer and the lower 10-bit PISO shift register. At the same time, the output of the upper comparator is also transferred to the upper 10-bit PISO shift register in preparation for providing the location and the defect type of the defective TSV. Using the 10-input AND gate, the test result analyzer consolidates all the enabled TSVs into a single result consisting of the concurrent TSV group; the result is either a pass or a fail. When a defective TSV is detected, the active low tri-state buffer with both the upper and lower 10-bit PISO shift register can classify the test result of each concurrent TSV block as a fault-free, resistive open defect or bridge defect. The upper 10-bit PISO shift register contains the defect information whether the TSV-to-TSV bridge defect occurred or not. The lower 10-bit PISO shift register provides the test result of each TSV, and it is used to activate or deactivate the tri-state buffer depending on the pass–fail result. If all TSVs of each concurrent TSV block pass the test, the pulse signal is transferred to the next flip-flop for turning on the other transistors to test the following TSV of each concurrent TSV block. In contrast, once the test-result analyzer detects a defective TSV, it activates the low tri-state buffer and both two 10-bit PISO shift registers by delaying the free-running clock (*Clk*) for entering the analysis mode until the consolidated result of the concurrent TSV group is entirely decompressed. Under this condition, the TSV test is paused for 10 test-clock-periods after the consolidated result of the concurrent test is sent to the pad, as shown in Fig 8.

To decompress the consolidated result of the concurrent TSV group, two 10-bit PISO shift registers are activated by automatically controlling the multiplexers. The output of the upper comparator is logic 0 (low) if the TSV has the TSV-to-TSV bridge defect. However, the output of the upper comparator is logic 1 (high) for both fault-free TSV and the TSV with a resistive open defect. In general, 20 test-clock-periods are required to classify the defect type among 10 TSVs of the concurrent test group in the analysis mode. The first 10 test-clock-periods are used to determine the location of the defective TSV in the concurrent test group (*N*_*Block*_ = 10); logic 1 is pass and logic 0 is fail. The defect type of the defective TSV can be determined between the TSV-to-TSV bridge defect and the resistive open defect during the next 10 test-clock-periods; logic 0 is the bridge fault and logic 1 is the open fault. To reduce the test time in half in the analysis mode, the active low tri-state buffer with the two 10-bit PISO shift registers is applied to the proposed concurrent TSV test architecture for strobing data into the shift register in parallel; the high impedance (i.e., Hi-Z) output can be enabled only in the analysis mode. The location information of the defective TSV is transferred to the lower shift register at the rising edge of the *ClkB*. The defect-type information of the defective TSV is also captured at the rising edge of the *ClkB*. Table 1 describes the active low tri-state buffer, whose output is always set to the Hi-Z status when the enabled signal is logic 1.

**Table 1:** Basic operation of the active low tri-state buffer.

Truth table		
Enable	Input	Output
Low	Low	Low
Low	High	High
High	Low	Hi-Z
High	High	Hi-Z

The test result of the defect detector is logic 1 if the tested TSV is fault free. For this reason, the output of the test result through the pad is the mid-band value for the fault-free TSV obtained by controlling the active low tri-state buffer. ATE supports the voltage termination (VT) mode to avoid voltage reflection; the user can program the voltage termination level for the output compare mode [32]. If the VT level is set to the midpoint value between the high and low output voltages of the device, the mid-band voltage level can be detected when the device output is in the Hi-Z state during this VT termination mode. Consequently, it is perfectly possible to distinguish the defect type of all defective TSVs in the concurrent test during the 10 test-clock-periods (Table 2). In the analysis mode, 10 results of a single TSV of each concurrent TSV block are sequentially sent to the pad through the active low tri-state buffer with two 10-bit PISO shift registers, which are synchronous with the test-clock-period (*ClkB*).

**Table 2:** Expected output of the proposed test architecture.

		Resistive open defects	TSV-to-TSV bridge defects	Fault-free
**Upper inverting comparator** **(bridge defect)**		High (Constant)	Low (Defective)	High (Indefective)
**Lower non-inverting comparator** **(open defect)**		Low (Defective)	High (Constant)	High (Indefective)
**Window comparator’s output** **(defect detector)**		Low (Defective)	Low (Defective)	High (Indefective)
**PAD**	**Normal** **mode**	Low (Fail)	Low (Fail)	High (Pass)
	**Analysis** **mode**	High (Open fault)	Low (Bridge fault)	Mid-band (Fault-free)

## Experimental results and analysis

In this section. we evaluate the proposed test architecture using a simulation experiment. As discussed in the previous section, the concurrent TSV test architecture proposed in this paper reduces the hardware overhead more than the previous test architectures. We compared the hardware size of the proposed test architecture with the previous test architectures by using the synthesis tool *Synopsys Design Vision* (Nangate 45nm Open Cell Library). To verify the functionality of the proposed concurrent TSV test architecture by using *HSPICE* (45nm gate library with predictive technology model (PTM) transistor models [36, 37]), we obtained the voltage profile of the TSVs from the observed TSV resistance changes. These simulation results are essential for determining the voltage reference of the comparator in the pass–fail limit. The TSV and FET specifications in the *HSPICE* simulation were extracted from published data [16, 17]. We set *R*_*TSV*_ = 2mΩ and C_*TSV*_ = 242fF. The width and length of the FETs were 8μm and 50nm, respectively, for pMOS, and 1μm and 50nm, respectively, for nMOS. Under these specifications, the voltages across each TSV and the peak current consumptions when the number of TSVs increased were also simulated by *HSPICE*. The total test time was further decreased from that of the previous test architectures. However, the test time of the proposed concurrent TSV test architecture is dependent on the number of defective TSVs. Therefore, we simulated the fault model experiment 10,000 times to obtain the test time.

### Simulation results of changing the TSV resistance

**Fig 9:**
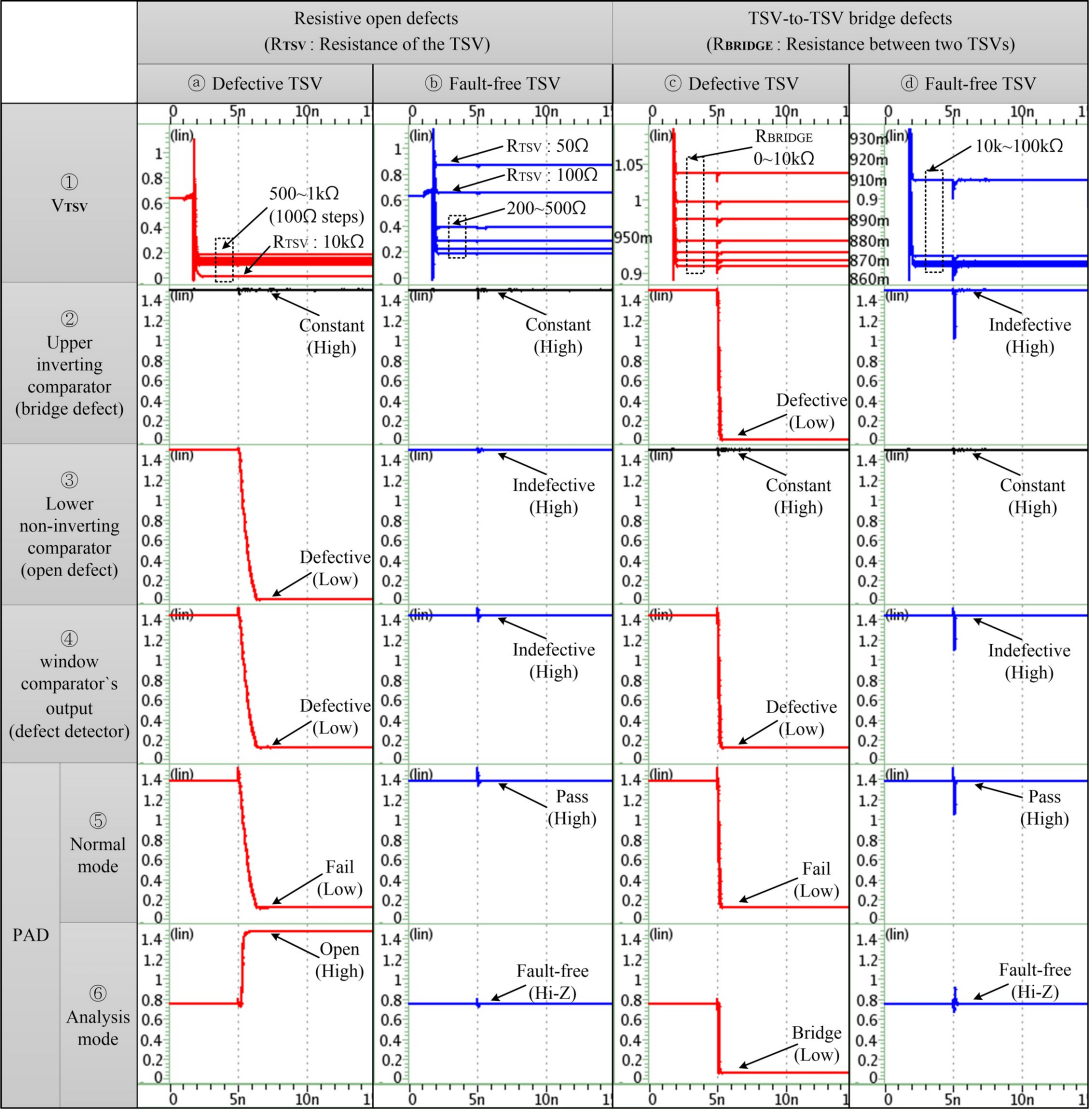
Voltage profiles as a function of *R*_*TSV*_ and *R*_*BRIDGE*_ changes.

As discussed in the previous section, each TSV of all the concurrent blocks requires the window comparator for the parallel detection of resistive open and bridge defects. Fig 9 ①–ⓐ, ①–ⓑ, ①–ⓒ and ①–ⓓ present the voltage profiles of the TSV of the proposed test architecture, which was obtained by varying *R*_*TSV*_ and *R*_*BRIDGE*_. The suitable reference voltage of the window comparators can be determined by changing *R*_*TSV*_ and *R*_*BRIDGE*_, and the reference input can be designed to supply from the device internally or externally. Let us assume that the failure conditions for the resistive open and TSV-to-TSV bridge defects occur when *R*_*TSV*_ and *R*_*BRIDGE*_ are above 500Ω and below 10kΩ, respectively. The output of the upper comparator (i.e., the inverting comparator circuit) was not correlated with *R*_*TSV*_ because this comparator was used to detect only the TSV-to-TSV bridge defect. Fig 9 ②–ⓐ and ②–ⓑ show that the output of the upper comparator remained in a constant high state regardless of *R*_*TSV*_. The bridge defect could be detected by monitoring the output of the upper comparator when *R*_*BRIDGE*_ was less than 10kΩ; the reference voltage of the upper comparator was 0.91V (Fig 9 ②–ⓒ and ②–ⓓ). The reference voltage of the lower comparator (i.e., the non-inverting comparator circuit) was set as 0.19V for detecting the resistive open defect. The output of the lower comparator in relation to *R*_*TSV*_ started to transit at 500Ω (Fig 9 ③–ⓐ and ③–ⓑ), but its output remained in a constant high state regardless of *R*_*BRIDGE*_ (Fig 9 ③–ⓒ and ③–ⓓ). The window comparator is implemented by using the upper and lower comparator as the defect detector, and it could simultaneously detect the resistive open and bridge defects. The output of the defect detector appeared in a low state when the TSV had any defect, such as the resistive open defect or the bridge defect (Fig 9 ④–ⓐ and ④–ⓒ). In contrast, it returned a high state under the fault-free TSV condition (Fig 9 ④–ⓑ and ④–ⓓ). Therefore, the resistive open defect could be detected by monitoring the output combinations of the upper comparator and the defect detector according to *V*_*TSV*_. Finally, the test result analyzer transferred the final test result by consolidating all the test results of the 10 defect detectors by using the 10-input AND gate. The final test result was the same as the consolidated output result of the defect detector under the normal mode (Fig 9 ⑤–ⓐ, ⑤–ⓑ, ⑤–ⓒ and ⑤–ⓓ). However, the final test result of the analysis mode through the pad could be changed by the active low tri-state buffer using the two 10-bit PISO shift registers. After the test result analyzer detected the defective TSV, the active low tri-state buffer was enabled on the next test cycle. Thus, the output was changed because of the following three levels under the analysis mode: high, low, and tri-state. The defect type of all defective TSVs can be classified by the changed result (as shown in Fig 9 ⑥–ⓐ and ⑥–ⓒ) as high (i.e., open defect) and low (i.e., bridge defect). The fault-free TSV returns the tri-state (mid-band) as the pass result (Fig 9 ⑥–ⓑ and ⑥–ⓓ). The functionality of the proposed test architecture was verified based on these *HSPICE* simulation results.

### Comparison of hardware overheads

**Fig 10:**
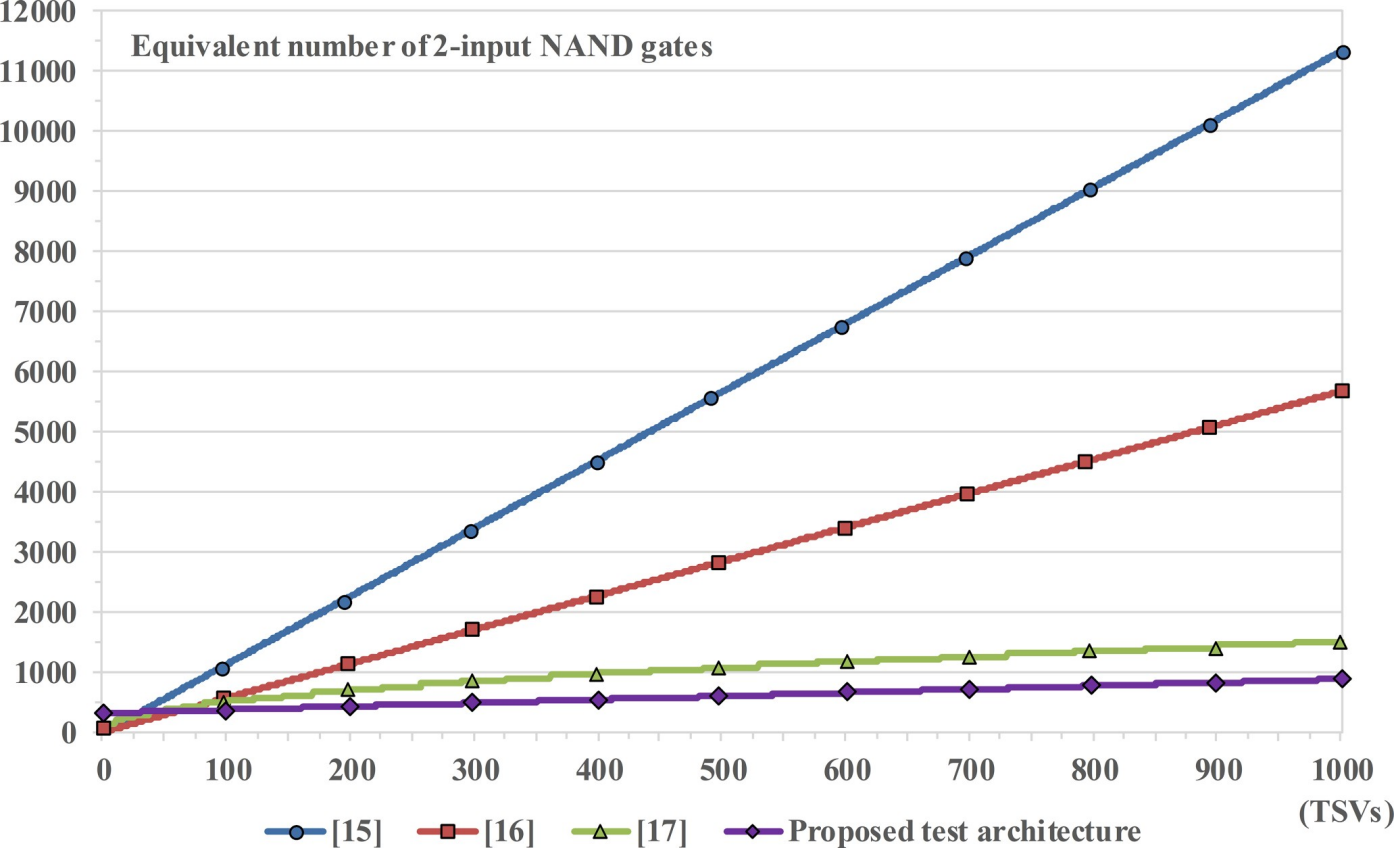
Comparison of total hardware sizes as functions of TSV number.

The total hardware size of the TSV test architectures linearly increases with the number of TSVs in 3D-ICs, and the required additional hardware size per TSV is different depending on the TSV test architectures. In this section, the hardware overhead of the proposed TSV test architecture is fairly compared with the previous works [15–17], which is basically synthesized by using the same standard cell library under the same conditions. The additional hardware overhead per TSV is represented as the number of components measured by the equivalent gate count. First, the number of voltage drivers was equal to *N*_*Block*_ and the number of voltage dividers was the same as that of all TSVs (i.e., *N*_*TSV*_). Second, the number of pulse-transfer flip-flops as the concurrent test controller was relative to *N*_*TSV_Block*_. The defect detector consists of window comparators, and its number was equal to *N*_*Block*_. Lastly, N-input AND gate in the test result analyzer and the N-bit PISO shift register in the output compression/decompression circuits were determined by *N*_*Block*_. Consequently, the hardware overhead of the proposed test architecture can be calculated as follows:

$$\begin{array}{l} Area_{total}=A_{1(voltage\;driver)}+A_{2(voltage\;divider)}+A_{3(concurrent\;test\;controller)}+A_{4(defect\;\mathrm{detector})}+A_{5(test\;result\;analyzer)}\\ \;+A_{6(test\;clock\;controller)}+A_{7(output\;compression\;and\;decompression\;circuits)} \end{array}$$ (3)

$$\begin{array}{l} Area_{total}=A_{1}[N_{Block}\times pMOS]+A_{2}[N_{TSV}\times (pMOS+nMOS+inverter)]+A_{3}[N_{\mathrm{TSV}\_\mathrm{Block}}\times flip‐flop]\\ \;+A_{4}[N_{Block}\times window\;comparator]+A_{5}[N_{\mathrm{Block}}‐input\;AND\;gate+flip‐flop+inverter]\\ \;+A_{6}[(2\times multiplexer)+inverter+flip‐flop]\\ \;+A_{7}[(2\times multiplexer)+(2\times N_{\mathrm{Block}}‐bit\;PISO)+flip‐flop+tri‐state\;buffer] \end{array}$$ (4)

where *A* denotes the area overhead of each main part; all other notations have their usual meanings. Fig 10 represents the synthesis results obtained as the equivalent gate count of the 2-input NAND gates determined by *Synopsys Design Vision* (Nangate 45nm Open Cell Library). The result compares the total hardware overhead (measured at the 2-input NAND gate) in the various architectures as functions of TSVs. The proposed test architecture provided less hardware overheads than the overheads in [15–17] having more than 26, 53, and 26 TSVs, respectively. It is fairly certain that there is an extremely low probability of using only these TSV numbers in real 3D-ICs. In case of 1,000 TSVs, the hardware overhead of the proposed test architecture decreased by 92.6%, 85.2%, and 43.8%, as compared to the overheads in [15–17], respectively. Consequently, the proposed test architecture has the lowest hardware overhead as compared with the TSV test architectures based on the voltage divider structure.

### Comparison of total execution test time

**Fig 11:**
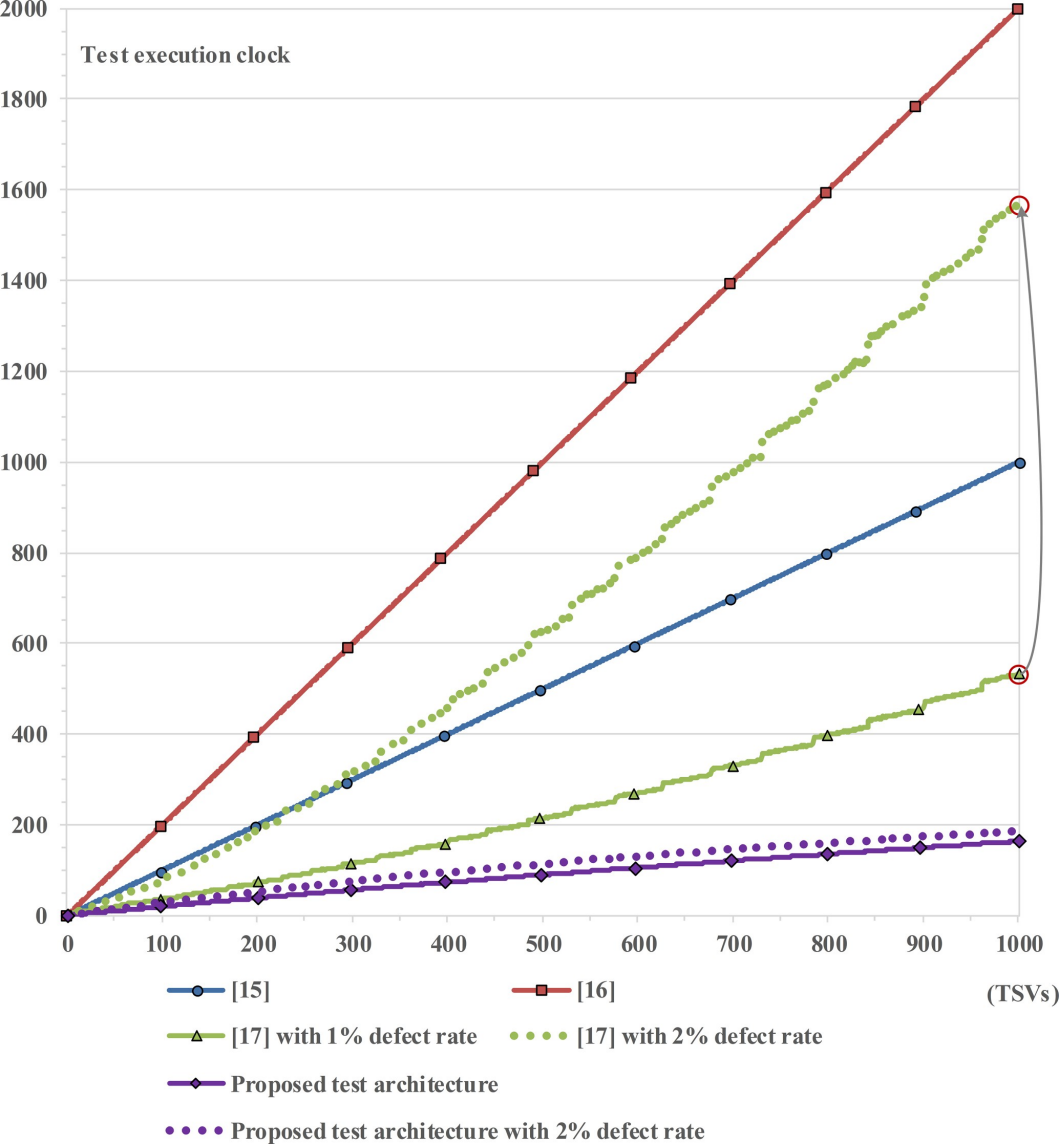
Comparison of total test times as functions of TSV number.

Fig 11 shows the comparison result of the total test time as functions of TSV numbers. To compare the test time, the expected test time was implemented by simulating the C-based faulty model experiment 10,000 times under a 0.01 defect probability condition; the open defect was 0.5% and the bridge defect was 0.5%, as mentioned in Section 3.2. In the previous works [15, 16], TSV testing took a long time because it increased in proportion to the number of TSVs. TSV testing in [17] was faster than that in [15, 16] by using the *n* × *n* matrix test method. However, we needed to classify the fault location and the defect type for maintaining the test quality when a defective TSV was detected. Therefore, the test time of [17] continued to increase by 2 × *n* test-clock-periods whenever the test result failed. The problem becomes more challenging when the n value is large and the number of TSVs in 3D-ICs has been rising continuously. In contrast, the proposed test architecture always requires 10 test-clock-periods for categorizing the fault location and the defect type at the same time, regardless of the number of TSVs, during the analysis mode. Moreover, the test time of the previous works [17] is affected by the TSV failure rate as mentioned in Section 3.1. As described in Fig 11, its test time is substantially increased for only 1% increase in the TSV failure rate; however, there is little or no effect of this failure rate on our test architecture.

In case of 1,000 TSVs, the total test time of the proposed test architecture under 1% probability of TSV defects decreased by 83.7%, 91.8%, and 69.4% as compared to the previous architectures in [15–17], respectively. As a result, the proposed test architecture can provide not only the lowest hardware overhead but also the fastest test time among the TSV test architectures based on the voltage divider structure.

### Comparison of peak current consumption

**Fig 12:**
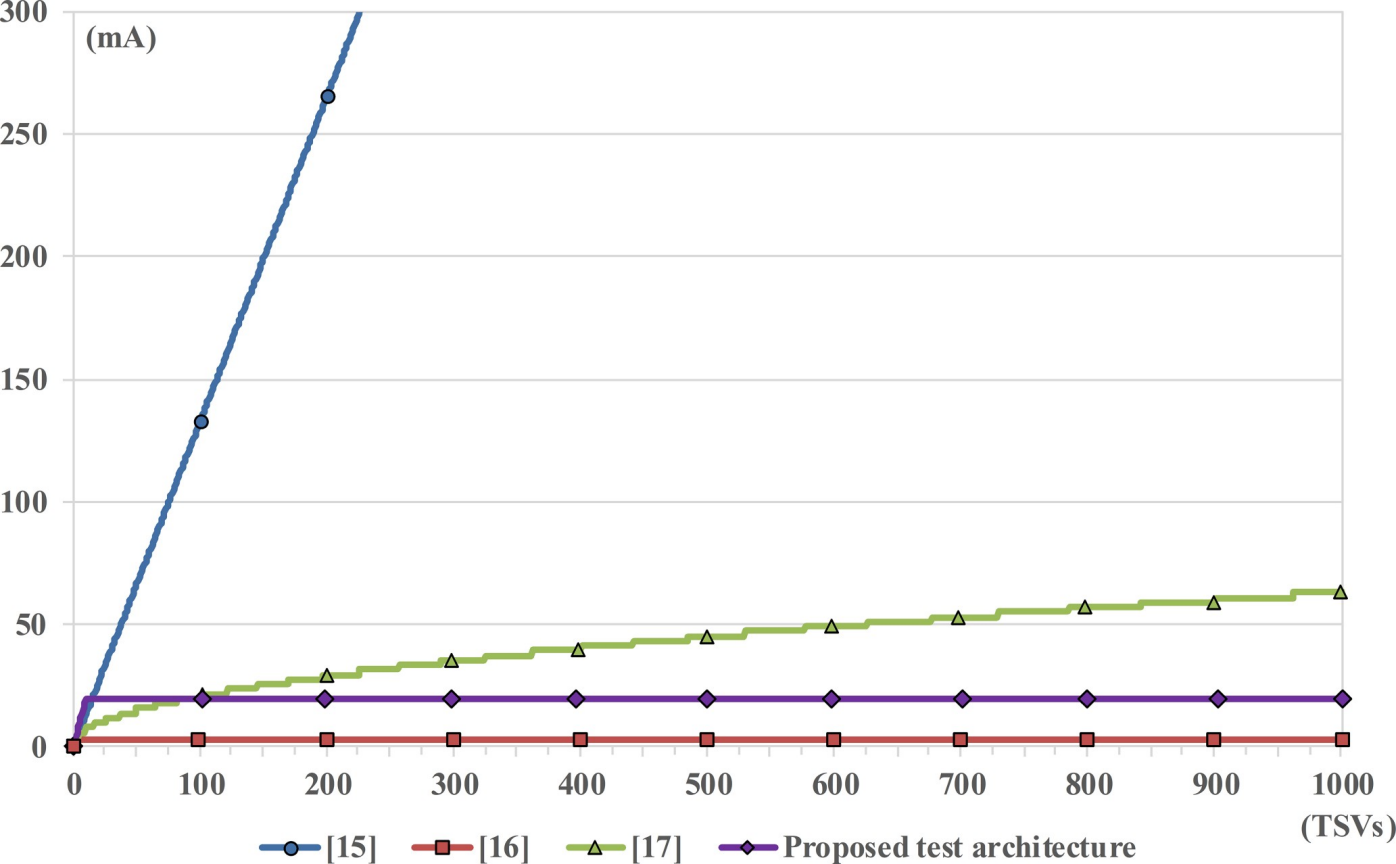
Comparison of peak current consumption as functions of TSV number.

The peak current consumption is measured when the direct current path from VDD to GND is produced by turning all voltage drivers and voltage dividers. For this reason, the peak current consumption is determined by the number of enabled TSVs for testing during the one test cycle period. In the previous work [15], all TSVs were simultaneously activated, which lead to a high peak current consumption. To overcome this drawback, a single TSV was tested at a time in [16]; this provided the lowest peak current consumption, as shown in Fig 12. In [17], the slight linear increase in the peak current consumption was provided under DPS constraints of ATE; this was relative to the square root of the number of TSVs. It is the appropriate value for mass production under *N*_*TSV*_ constraints, but the peak current consumption in [17] can exceed the DPS constraints at any moment if *N*_*TSV*_ continues to increase. Finally, the proposed test architecture always enables a single TSV of each concurrent test block (a total of 10 TSVs) for each test cycle period. According to the *HSPICE* results, the peak current consumption of the proposed concurrent TSV test architecture appears like a slightly incremented value for mass production as compared with [16]. However, it is actually still a reasonable value for mass production because the high pin count DPS can generally provide maximum current outputs up to 800 mA as mentioned in Section 3.2. Consequently, both [16] and the proposed test architecture can also be applied to the low-end ATE because both architectures can steadily provide very low fixed-peak current consumption, regardless of the number of TSVs. The architecture in [16] and our proposed architecture can provide a consumption of 3.28 mA and 19.65 mA, respectively.

### Summary comparison of test architectures

**Fig 13:**
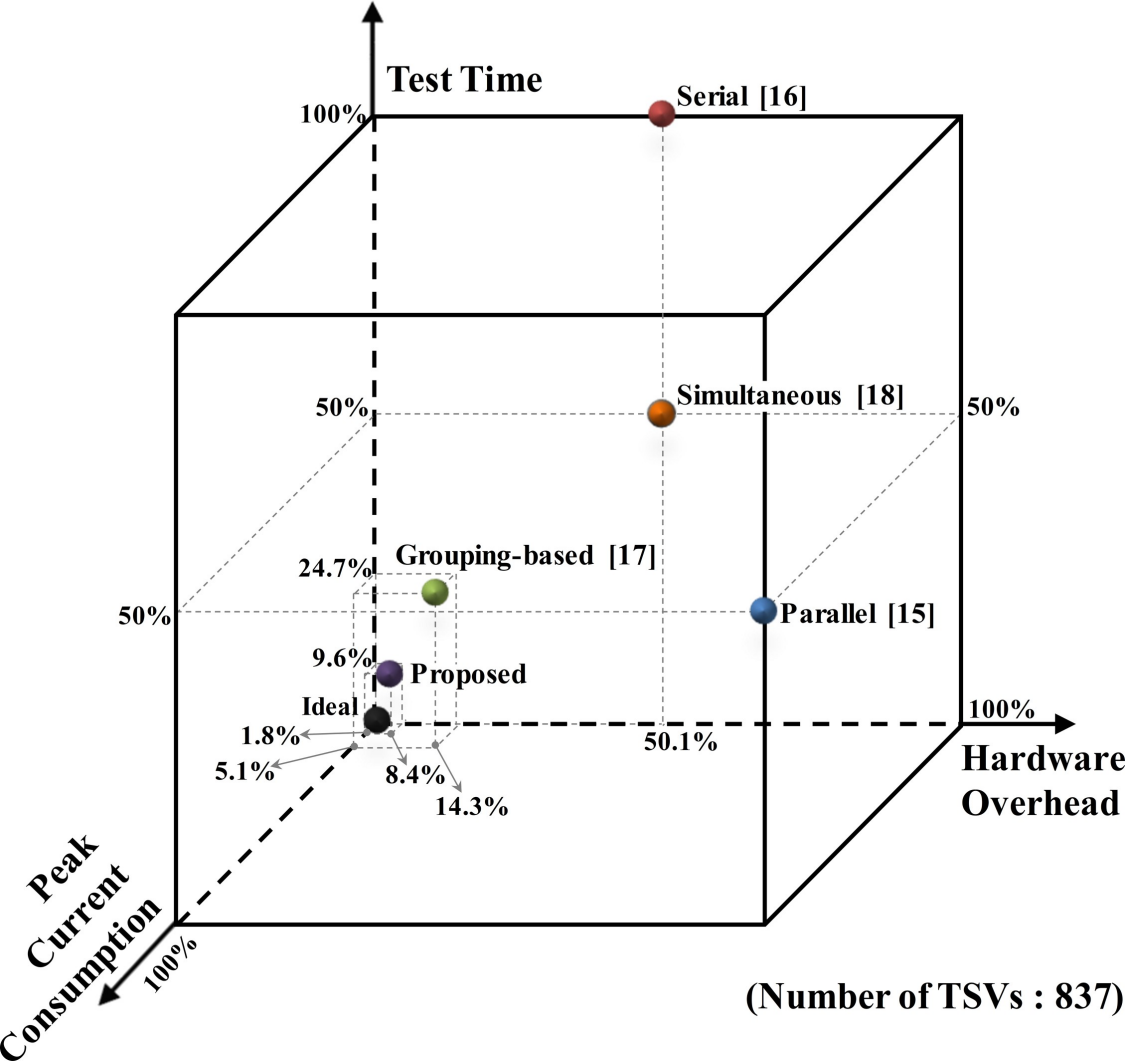
Comparison graph of test architectures.

S1 File and Fig 13 represent the comparison graph with specific attributes such as hardware overhead, test time, and peak current consumption, when applying the previous and proposed test architectures to the actual AES device with 837 TSVs. The parameters are expressed as ratios of the corresponding parameters in the worst results among the TSV test architectures based on the voltage divider structure. The test time of [15] includes only the test times of the resistive open defects because the TSV-to-TSV bridge defects cannot be detected in this architecture. In the test time column, the times in parentheses indicate the total times in [16], which cannot detect the resistive open and bridge defects in parallel. In contrast, in [18], the resistive open and bridge defects for reducing the test time in half can be tested simultaneously. In [17], the test time can be drastically reduced using the n × n matrix test method. However, the proposed test architecture is faster than any previous TSV test architecture, and it does not compromise on the test quality. Consequently, we prove that the proposed test architecture is a more effective solution than the previous test architectures. The proposed architecture can also reduce the cost of testing TSVs in 3D-ICs by significantly reducing the test time and the hardware overheads and by providing reasonable peak power consumption for mass production.

## Conclusions

It is important for semiconductor companies to reduce their manufacturing cost for mass production. In case of TSV-based 3D-ICs, the manufacturing cost can be decreased by reducing the test time or hardware overhead. Our proposed concurrent TSV test architecture can be a solution to the problems related to high manufacturing costs. This architecture allows us to reduce the test time more efficiently. Unlike other test architectures, the proposed test architecture effectively reduces both the total test time and the hardware overhead without compromising on the test quality. The increase in the test times and hardware overheads as functions of the TSV number is much slower in our proposed test architecture as compared with the increase in the previous architectures based on the voltage divider structure. This proves the significant advantage of our proposed architecture, especially when testing a large number of TSVs. The proposed test architecture is strongly supported by the experimental results based on the actual TSV distribution of the AES device having 837 TSVs as the sample device. Therefore, we proved that our proposed architecture reduces the TSV test cost of 3D-ICs for mass production by decreasing the test time and hardware overhead while ensuring appropriate peak current consumption.

## Supporting information

S1 File Performance comparisons of test architectures. The excel document contains the specific numbers and results in the comparison graph with specific attributes such as hardware overhead, test time, and peak current consumption. Every sheet shows the detailed comparison results of the hardware overhead, test time, and peak current consumption as functions of TSV numbers (0 to 1000), respectively.
(XLSX)
